# Mechanism of KDM5C‐Mediated H3K4me3 Demethylation of HOXC‐AS3 in the Proliferation of Colorectal Cancer Cells

**DOI:** 10.1002/kjm2.70068

**Published:** 2025-07-08

**Authors:** Hong Li, Da‐Min Li, Zhan Wang, Jie Hou

**Affiliations:** ^1^ Department of Gastrointestinal and Anal Surgery People's Hospital of Baoan District Shenzhen Guangdong China

**Keywords:** colorectal cancer, DLG4, HOXC‐AS3, KDM5C, YTHDC1

## Abstract

Colorectal cancer (CRC) ranks as the third most prevalent malignancy and the second leading cause of cancer‐related mortality worldwide. This study explored the role of lysine‐specific histone demethylase 5C (KDM5C) in CRC progression. Expression levels of KDM5C, HOXC‐AS3, and discs large MAGUK scaffold protein 4 (DLG4) were evaluated. Following the KDM5C knockdown, cell proliferation assays were conducted. The recruitment of KDM5C and histone H3 lysine 4 tri‐methylation (H3K4me3) to the HOXC‐AS3 promoter region was investigated. Furthermore, the subcellular distribution of HOXC‐AS3 was assessed using nuclear‐cytoplasmic fractionation and RNA fluorescence in situ hybridization (RNA‐FISH). The interactions between HOXC‐AS3, YTH domain‐containing protein 1 (YTHDC1), and DLG4 were detected. The stability of DLG4 mRNA was evaluated, and the functional roles of HOXC‐AS3 and DLG4 in CRC cells were examined through combined experimental analyses. KDM5C expression was elevated in CRC cells, whereas HOXC‐AS3 and DLG4 levels were notably reduced. Silencing KDM5C resulted in suppressed cell proliferation. Mechanistically, KDM5C inhibited HOXC‐AS3 expression by demethylating H3K4me3 at its promoter. HOXC‐AS3 promoted DLG4 mRNA stability by recruiting the RNA‐binding protein YTHDC1. Combined experimental results indicated that overexpression of HOXC‐AS3 or DLG4 reduced the inhibitory effect of KDM5C downregulation on CRC cells. In conclusion, KDM5C promotes CRC cell proliferation by demethylating H3K4me3, repressing HOXC‐AS3 expression. The reduced HOXC‐AS3 levels impair the recruitment of YTHDC1, leading to decreased DLG4 expression.

## 1 Introduction

Colorectal cancer (CRC) is the third most commonly diagnosed malignancy and the second leading cause of cancer‐related mortality worldwide, contributing to approximately 10% of all cancer cases and 9.4% of cancer deaths globally [1]. The pathophysiology of CRC primarily involves abnormal cell proliferation, differentiation, and resistance to apoptosis, which are largely influenced by genetic and environmental factors [2]. Although screening and early interventions have effectively reduced CRC mortality rates, the survival for advanced diseases remains dismal [3]. Although current treatment options for CRC—such as biological therapy, immunotherapy, combination regimens, and surgical resection—are widely employed, patient survival rates remain suboptimal [4]. Therefore, advancing early diagnostic strategies and developing more effective interventions, particularly for advanced‐stage CRC, remain critical priorities.

Lysine‐specific histone demethylase 5C (KDM5C) is a member of the KDM protein family and acts as a regulatory factor involved in the removal of histone H3 lysine 4 dimethylation and trimethylation (H3K4me2/me3) [5]. Extensive studies indicate that KDM5C is an oncogenic factor in several malignancies, including prostate, bladder, and colon cancers [6, 7, 8]. H3K4me3 is predominantly enriched at gene promoter regions, modulating gene expression and chromatin architecture. Its dysregulation contributes to epigenetic alterations involved in tumor initiation and progression. In CRC, KDM5C is highly overexpressed in cell lines and clinical animal models, facilitating the demethylation of H3K4me3 [9]. KDM5C‐mediated demethylation of H3K4me3 inhibits the expression of downstream factors, thus promoting tumor metastasis in CRC [10]. However, the downstream mechanisms by which KDM5C influences CRC through H3K4me3 demethylation remain to be fully clarified.

Long non‐coding RNAs (lncRNAs) are RNA transcripts longer than 200 nucleotides that do not encode proteins and are considered functional units [11]. HOXC‐AS3 is a recently identified lncRNA that is aberrantly expressed in multiple cancers, with its upregulation closely linked to key cellular processes, including cell proliferation, apoptosis, invasion, metastasis, and metabolic reprogramming [12]. A previous study has confirmed that HOXC‐AS3 is significantly downregulated in CRC tissues and is associated with poor survival rates [13]. On the other hand, a decrease in H3K4me3 levels at the HOXC‐AS3 promoter in non‐small cell lung cancer effectively suppresses HOXC‐AS3 expression [14]. This study hypothesizes that HOXC‐AS3 may be regulated by KDM5C‐mediated H3K4me3 demethylation in CRC progression.

In preliminary studies, database analysis suggested that HOXC‐AS3 may recruit YTHDC1 to stabilize DLG4 mRNA. YTHDC1, an N6‐methyladenosine (m6A) binding protein, recognizes m6A modifications on target genes and regulates their expression [15]. YTHDC1 acts as an RNA‐binding protein that interacts with lncRNAs to increase the stability of downstream mRNA, accelerating CRC progression [16]. Extensive evidence suggests that lncRNAs may regulate DLG4 within cancer regulatory networks [17, 18]. Notably, DLG4 is downregulated in CRC and is inversely correlated with cancer cell proliferation [19].

In this study, the role of the KDM5C/HOXC‐AS3/DLG4 axis in CRC was investigated. This study also investigated the effects of KDM5C on CRC cell proliferation to elucidate a novel epigenetic mechanism contributing to CRC progression and to identify potential targets for precision therapy.

## 2 Materials and Methods

### 2.1 Cell Culture

Human CRC cell lines HCT116 and SW480 were sourced from the American Type Culture Collection (Manassas, VA, USA). The normal colon cell line NCM460 was obtained from Shanghai Eco Biotechnology Co. Ltd. (Shanghai, China). Cells were cultured in Dulbecco's modified Eagle medium (DMEM; Thermo Fisher Scientific, Waltham, MA, USA) supplemented with 10% fetal bovine serum (FBS) and maintained in a humidified incubator at 37°C with 5% CO_2_.

### 2.2 Cell Treatment and Transfection

The YTHDC1 expression vector was constructed using the pcDNA3.1 vector (Sangon, Shanghai, China) to modulate YTHDC1 expression. KDM5C, HOXC‐AS3, DLG4 siRNAs, and negative control (NC) siRNA were purchased from Invitrogen (Shanghai, China). CRC cells were transfected with the appropriate constructs using Lipofectamine 3000 (Invitrogen, Carlsbad, CA, USA) when they reached 70%–80% confluence. A total of 1 × 10^6^ cells were transfected with 10 μM of the vector or 50 nM of siRNA. Experiments were performed 24 h post‐transfection.

### 2.3 Cell Proliferation

Cell proliferation viability was detected using the cell counting kit‐8 (CCK‐8) (Dojindo Laboratories, Kumamoto, Japan) based on the chemical reaction of tetrazolium salt (WST‐8). Briefly, CRC cells were seeded into 96‐well plates at 5000 cells per well with three replicates per group and incubated at 37°C and 5% CO_2_ for 24, 48, and 72 h. After incubation, 10 μL of CCK‐8 solution was added to each well for 2 h at 37°C. Absorbance was measured at 450 nm using a microplate reader (Bio‐Rad Laboratories, Hercules, CA, USA).

The colony formation assay is used to assess in vitro cell survival capacity based on the cells' ability to proliferate and differentiate. For this assay, 5 × 10^2^ CRC cells were seeded into six‐well plates and cultured for 14 days. Colonies were fixed with formaldehyde and stained with 0.1% crystal violet. After washing with phosphate‐buffered saline (PBS), colonies containing more than 50 cells were counted and photographed.

### 2.4 Chromatin Immunoprecipitation (ChIP)

Chromatin immunoprecipitation is based on the ability to recognize and bind the complex formed between a target protein and DNA using specific antibodies, co‐precipitating the DNA fragments interacting with the protein. A ChIP assay kit (Beyotime, Shanghai, China) was used to evaluate the binding of KDM5C/H3K4me3 to the HOXC‐AS3 promoter, following the manufacturer's instructions. Briefly, CRC cells were cross‐linked with 1% formaldehyde, and DNA fragments were generated by sonication on ice. The resulting samples were incubated overnight at 4°C with anti‐KDM5C (NB100‐55327, Novus Biologicals, Littleton, CO, USA), anti‐H3K4me3 (ab8580, Abcam, Cambridge, MA, USA), or control immunoglobulin G (IgG; ab171870, Abcam). The immunoprecipitated complexes were then eluted and de‐crosslinked overnight using NaCl solution. Chromatin DNA was recovered from the precipitates, dissolved in deionized water, and analyzed by quantitative polymerase chain reaction (qPCR).

### 2.5 Nuclear and Cytoplasmic Fraction Assay

The cytoplasmic and nuclear fractions of CRC cancer cells were isolated using the Cytoplasmic and Nuclear RNA Purification Kit (Norgen Biotek Corp, Thorold, ON, Canada). Briefly, CRC cells were lysed on ice for 15 min. Following centrifugation, nuclear and cytoplasmic fractions were collected separately. RNA was extracted from both fractions using TRIzol reagent (Invitrogen) and subjected to RT‐qPCR. U6 was a nuclear control, and Glyceraldehyde‐3‐phosphate dehydrogenase (GAPDH) was a cytoplasmic control.

HOXC‐AS3‐specific probes were synthesized by GenePharma (Shanghai, China). Briefly, CRC cells were seeded onto confocal dishes (Nest Scientific, Woodbridge, NJ, USA), washed, fixed, and permeabilized with 0.1% Triton X‐100. Cells were pre‐hybridized for 40 min for probe detection, followed by overnight incubation with specific probes at 37°C. After washing with hybridization buffer, cells were stained with 4′,6‐diamidino‐2‐phenylindole (DAPI) for 30 min at 37°C. Fluorescent images were captured using a Dragonfly 200 high‐speed confocal microscope (Andor Technology, Belfast, UK).

### 2.6 Bioinformatics Analysis

The STARbase database (https://rnasysu.com/encori/) [20], RNAInter database (http://www.rna‐society.org/rnainter/) [21], and RBPDB database (http://rbpdb.ccbr.utoronto.ca/) [22] were used to predict proteins that may interact with HOXC‐AS3. Moreover, the STARbase database predicted mRNAs that can potentially bind to YTHDC1.

### 2.7 RNA Immunoprecipitation (RIP)

The principle of RIP is to capture endogenous RNA‐binding proteins using specific antibodies and isolate these proteins along with their bound RNAs via immunoprecipitation. The EZ‐Magna RIP kit (MilliporeSigma, Burlington, MA, USA) performed RIP analysis. Briefly, CRC cells were incubated on ice for 60 min with RIP lysis buffer containing RNase and protease inhibitors. The cell lysates were then incubated overnight with magnetic beads conjugated to YTHDC1 antibody (ab264375, Abcam) or control IgG (ab171870, Abcam). The immunocomplexes were washed five times and treated with proteinase K. Immunoprecipitated RNA was subsequently isolated for RT‐qPCR analysis.

### 2.8 RNA Pull‐Down Assay

The principle of RNA pull‐down is to use labeled RNA probes to bind target proteins, followed by isolation of the resulting complexes through techniques such as co‐immunoprecipitation. Biotinylated HOXC‐AS3 and NC probes were designed and synthesized by GenePharma (Shanghai, China). Briefly, CRC cells were fixed and lysed in a lysis buffer. After centrifugation, the supernatant was collected, and 50 μL was set aside as the input sample while the remaining lysates were incubated with the biotin‐labeled probes. The mixture was then incubated overnight with DynaBeads conjugated to streptavidin. The beads capturing the immunocomplexes were washed twice and eluted with buffer. Protein detection was carried out using western blot analysis.

### 2.9 Actinomycin D Treatment

In the actinomycin D (inhibits RNA synthesis) assay, CRC cells were incubated with 5 μg/mL actinomycin D (Sigma‐Aldrich, St. Louis, MO, USA) for designated periods. RNA was extracted at each time point for RT‐qPCR analysis.

### 2.10 RT‐qPCR

The principle of RT‐qPCR is based on DNA polymerase's ability to synthesize new DNA strands during PCR, coupled with fluorescently labeled probes or dyes to monitor the progression of the reaction through real‐time detection of increasing fluorescence. Total RNA was extracted from cells using TRIzol reagent (Invitrogen), and all RNA samples were treated with DNase I to remove any genomic DNA. Complementary DNA (cDNA) was synthesized from the RNA using the PrimeScript RT Reagent Kit (Takara, Tokyo, Japan). qPCR mixtures were prepared using the QuantiFast SYBR Green PCR Kit (Qiagen, Shanghai, China), with cDNA as the template. GAPDH was employed as the internal reference. The relative expression levels were calculated using the 2^−ΔΔCt^ method [23]. The primers are listed in Table 1.

**TABLE 1:** PCR primer sequences.

Genes	Accession numbers	Sequences (5′–3′)
KDM5C	NM_001146702	F: CTGGCCCTGTGTTGGAACT
		R: GGACTCAGGGATGCTGTGGT
HOXC‐AS3	NR_047506	F: GTGGAGTAACAGCGCCATCT
		R: CGGGTTTTGTTGCGTCTTGT
DLG4	NM_001128827	F: TGACAACCAAGAAATACCGCTAC
		R: GTCCCGTTCACATATCCTGG
YTHDC1	NM_001031732	F: CAGGAAGTGGACAGACGATT
		R: GTGGTGGTGGTCCCATGTTA
HOXC‐AS3 promoter	NR_047506	F: CCAAATGCCCTGTTCCAACG
		R: TGCGAAACGCGATTTGTTGT
GAPDH	NM_001256799	F: GATGCTGGCGCTGAGTACG
		R: GCTAAGCAGTTGGTGGTGC

### 2.11 Western Blot Assay

Western blot analysis is based on the specific binding between antigens and antibodies. This study extracted total protein from cells using radioimmunoprecipitation assay (RIPA) buffer. The proteins were then separated by 12% sodium dodecyl sulfate‐polyacrylamide gel electrophoresis (SDS‐PAGE) following denaturation in boiling water for 5 min. After separation, the proteins were transferred to a polyvinylidene fluoride (PVDF) membrane and blocked with 5% non‐fat milk at room temperature for 2 h. The membrane was incubated overnight at 4°C with primary antibodies against KDM5C (ab264168, 1:2000, Abcam), YTHDC1 (ab264375, 1:2000, Abcam), DLG4 (ab238135, 1:2000, Abcam), and β‐actin (ab5694, 1:1000, Abcam). The membrane was then incubated with a secondary antibody (ab205718, 1:2000, Abcam) at room temperature for 2 h. The bands were visualized using an enhanced chemiluminescence kit (Millipore, Billerica, MA, USA). The grayscale values of the Western blot results were quantified using Image J Software. The ratio of the grayscale value of the target protein to that of β‐actin was calculated, and protein expression was evaluated.

### 2.12 Statistical Methods

Statistical analysis and data visualization were performed using SPSS 21.0 software (IBM, Armonk, NY, USA) and GraphPad Prism 8.0 software (GraphPad Software, San Diego, CA, USA). The data's normality and homogeneity of variance were first assessed, confirming a normal distribution and homogeneity. Differences between the two groups were compared using the *t*‐test. However, comparisons among multiple groups were conducted using one‐way or two‐way analysis of variance (ANOVA), followed by Tukey's multiple comparisons test. All tests were two‐tailed. A *p*‐value of < 0.05 was considered statistically significant, while a *p*‐value of < 0.01 was regarded as highly statistically significant.

## 3 Results

### 3.1 KDM5C is Overexpressed in CRC and KDM5C Downregulation Inhibits CRC Cell Proliferation

**FIGURE 1:**
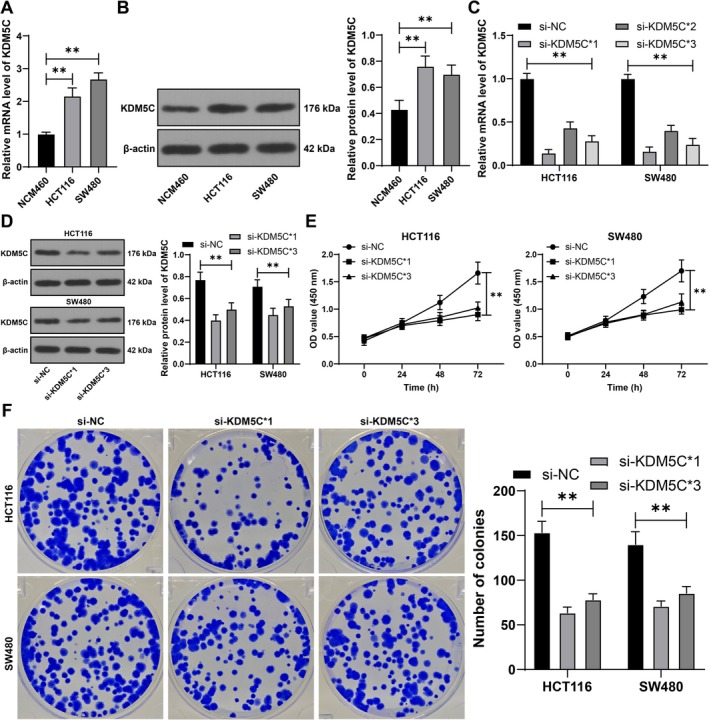
Overexpression of KDM5C in CRC, inhibition of CRC cell proliferation through KDM5C downregulation. (A, B) RT‐qPCR and Western blot were used to detect the expression of KDM5C in different cells. KDM5C siRNA (si‐KDM5C) was transfected into HCT116 and SW480 cells, with transfection of NC siRNA (si‐NC) as a negative control. (C, D) RT‐qPCR and Western blot were used to detect KDM5C expression in cells. (E) Cell proliferation was assessed using CCK‐8 assay. (F) Colony formation assay was performed to assess cell proliferation. The experiments were independently repeated three times, and the data are presented as the mean ± standard deviation. Multiple group comparisons in panel A–B were analyzed using one‐way ANOVA. In comparison, multiple group comparisons in panels C–F were analyzed using two‐way ANOVA, followed by Tukey's multiple comparisons test. **p < 0.01.

KDM5C expression is elevated in CRC [9]. To explore the impact and mechanism of KDM5C on CRC cell proliferation, NCM460 cells were cultured along with CRC cell lines. The results revealed an evident increase in KDM5C expression in HCT116 and SW480 cells compared to NCM460 cells (*p* < 0.01, Figure 1A,B). Subsequently, KDM5C expression was knocked down in cells through siRNA transfection (*p* < 0.01, Figure 1C,D), and two siRNAs with better transfection efficiency were selected to validate the effect of KDM5C on CRC cell proliferation. The proliferation of HCT116 and SW480 cells was decreased, and colony formation was inhibited after the downregulation of KDM5C (*p* < 0.01, Figure 1E,F). These results indicate that KDM5C is overexpressed in CRC cells, and KDM5C downregulation inhibits CRC cell proliferation.

### 3.2 KDM5C‐Mediated H3K4me3 Demethylation Suppresses HOXC‐AS3 Expression

**FIGURE 2:**
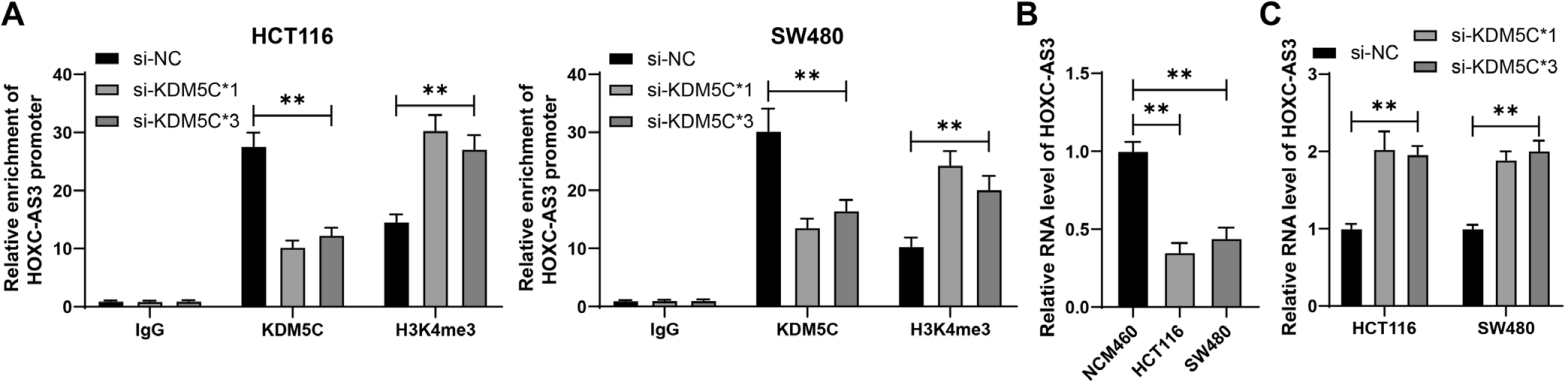
Suppresses of HOXC‐AS3 expression through KDM5C‐mediated H3K4me3 demethylation. (A) The enrichment of KDM5C and H3K4me3 on the HOXC‐AS3 promoter was analyzed by ChIP. (B, C) RT‐qPCR was used to detect HOXC‐AS3 expression in cells. The experiments were independently repeated three times, and the data are presented as the mean ± standard deviation. Multiple group comparisons in panel B were analyzed using one‐way ANOVA. Multiple group comparisons in panels A and C were analyzed using two‐way ANOVA, followed by Tukey's multiple comparisons test. **p < 0.01.

Previous studies have shown that KDM5C‐mediated H3K4me3 demethylation on the METTL14 promoter can inhibit METTL14 transcription [10]. Furthermore, HOXC‐AS3 is significantly downregulated in CRC tissues [13]. It was hypothesized that HOXC‐AS3 might be a downstream target of KDM5C. ChIP analysis revealed the enrichment of KDM5C and H3K4me3 on the HOXC‐AS3 promoter. After KDM5C knockdown, KDM5C enrichment was reduced, while H3K4me3 enrichment increased (*p* < 0.01, Figure 2A). HOXC‐AS3 expression was decreased in HCT116 and SW480 cells compared to that in NCM460 cells (*p* < 0.01, Figure 2B), and HOXC‐AS3 expression was increased in the si‐KDM5C group compared to that in the si‐NC group (*p* < 0.01, Figure 2C). In summary, KDM5C‐mediated H3K4me3 demethylation suppresses HOXC‐AS3 expression.

### 3.3 Downregulation of HOXC‐AS3 Alleviates the Inhibitory Effect of KDM5C Downregulation on CRC Cell Proliferation

**FIGURE 3:**
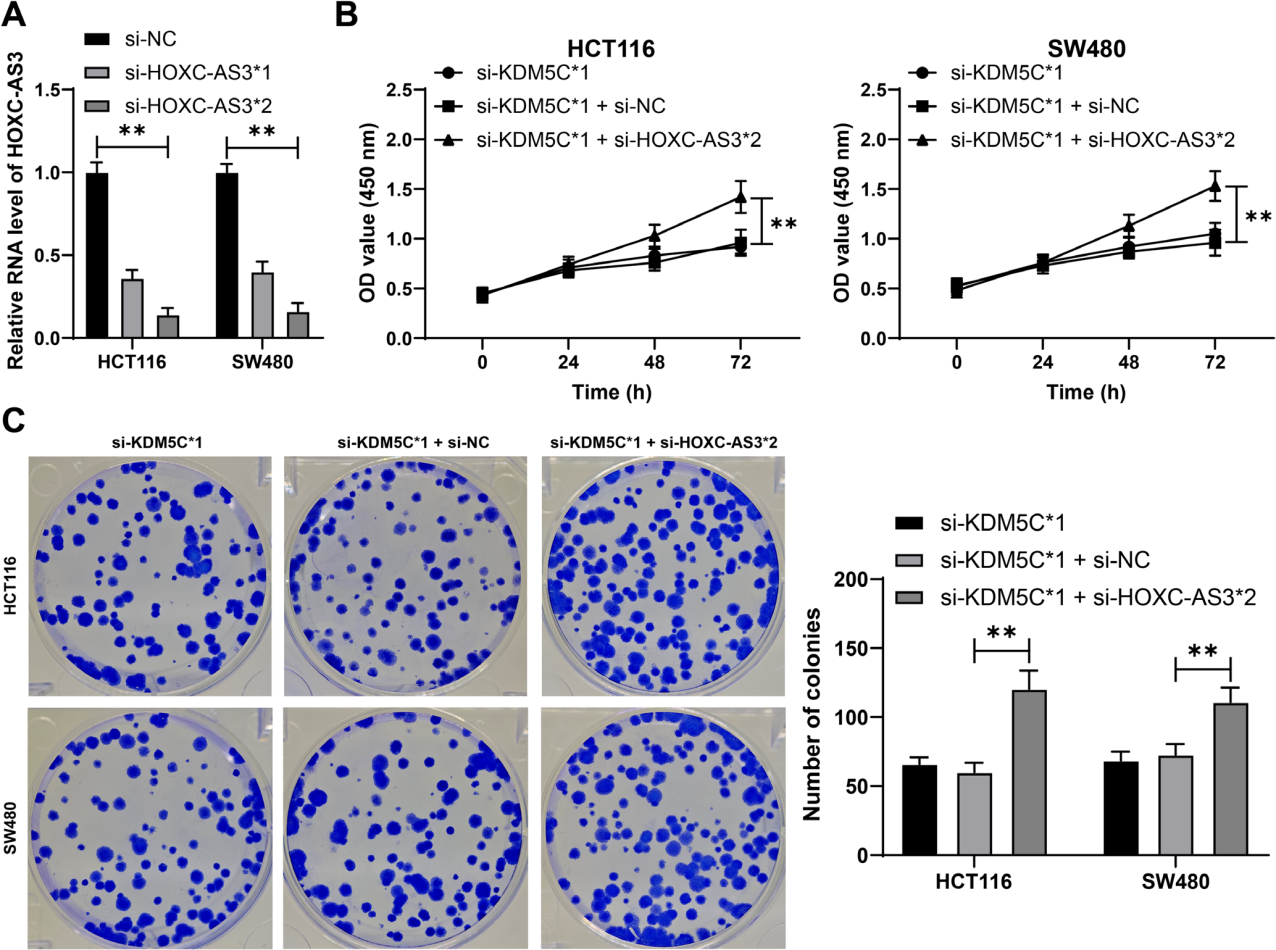
Alleviation of the inhibitory effect of KDM5C downregulation on CRC cell proliferation through downregulation of HOXC‐AS3. HOXC‐AS3 siRNA (si‐HOXC‐AS3) was transfected into HCT116 and SW480 cells, with transfection of NC siRNA (si‐NC) as a negative control. (A) RT‐qPCR was used to detect HOXC‐AS3 expression in cells. (B) Cell proliferation was assessed using CCK‐8 assay. (C) Colony formation assay was performed to assess cell proliferation. The experiments were independently repeated three times, and the data are presented as the mean ± standard deviation. Multiple group comparisons in panels A–C were analyzed using two‐way ANOVA, followed by Tukey's multiple comparisons test. **p < 0.01.

The HOXC‐AS3 expression in cells was then downregulated and combined experiments were carried out using si‐HOXC‐AS3*2 and si‐KDM5C*1, which showed improved transfection efficiency (*p* < 0.01, Figure 3A). Following the downregulation of HOXC‐AS3, cell proliferation was increased (*p* < 0.01, Figure 3B), and colony formation was promoted in cells (*p* < 0.01, Figure 3C). These results indicate that the downregulation of HOXC‐AS3 alleviates the inhibitory effect of KDM5C downregulation on CRC cell proliferation.

### 3.4 HOXC‐AS3 Recruits YTHDC1 to Promote DLG4 Expression

**FIGURE 4:**
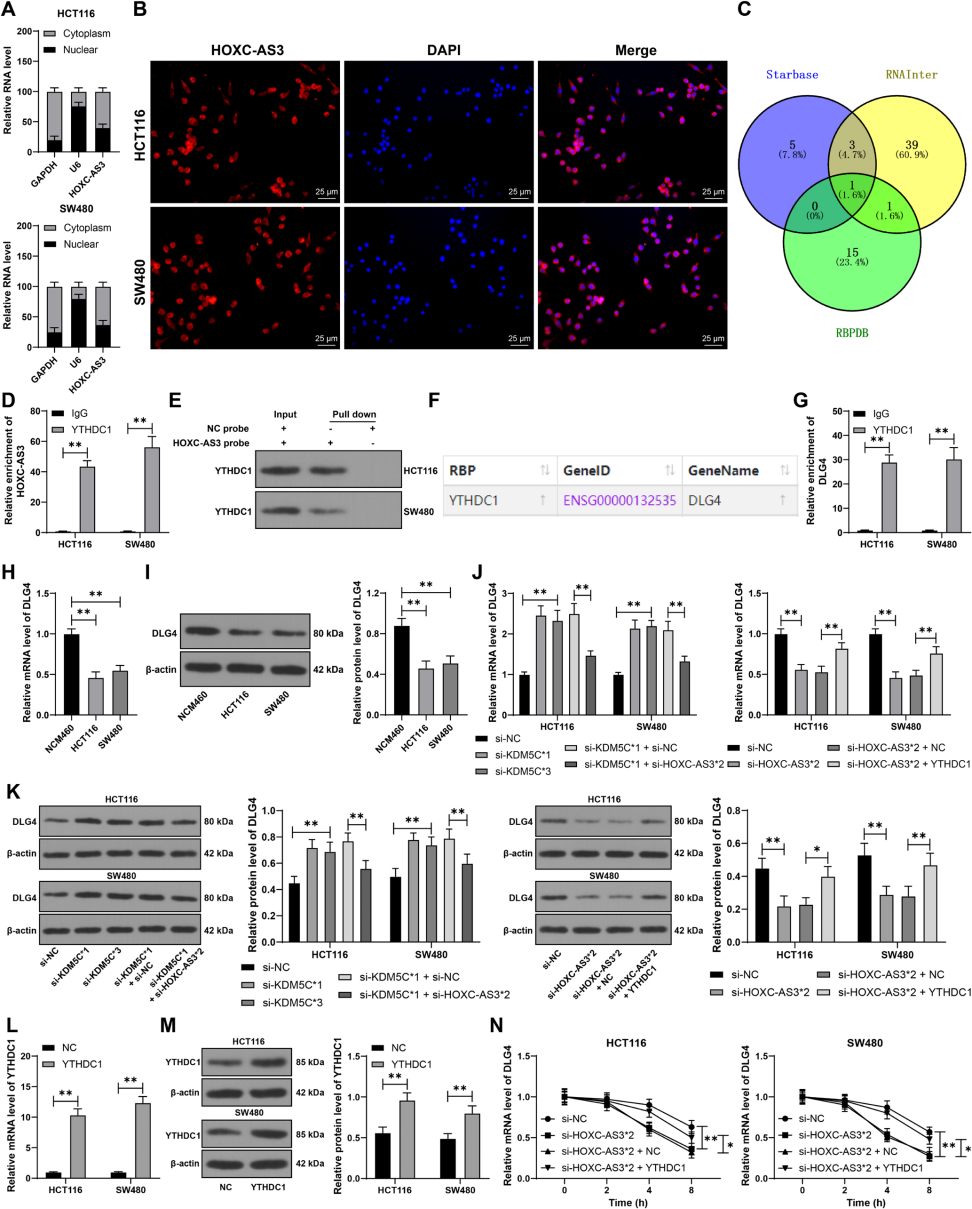
Recruitment of YTHDC1 by HOXC‐AS3 and promotion of DLG4 expression. (A, B) Subcellular fractionation and RNA FISH were performed to analyze the subcellular localization of HOXC‐AS3. (C) Multiple databases were used to predict proteins that can bind to HOXC‐AS3, and the intersection was taken. (D, E) RIP and RNA pull‐down were used to analyze the binding of HOXC‐AS3 with YTHDC1. (F) Interaction of YTHDC1 with DLG4 mRNA was predicted. (G) RIP was performed to analyze the binding of YTHDC1 to DLG4 mRNA. (H–K) RT‐qPCR and Western blot were used to detect the DLG4 expression in different cells. (L, M) RT‐qPCR and Western blot were used to detect the transfection efficiency of YTHDC1 in cells; (N) After treatment with actinomycin D, RT‐qPCR was used to detect DLG4 expression in cells. The experiments were independently repeated three times, and the data are presented as the mean ± standard deviation. Multiple group comparisons in panels H–I were analyzed using one‐way ANOVA. In comparison, multiple group comparisons in panels D, G, and J–N were analyzed using two‐way ANOVA, followed by Tukey's multiple comparisons test. *p < 0.05, **p < 0.01.

Following this, the molecular mechanism involving HOXC‐AS3 was further investigated. Subcellular fractionation and RNA FISH assays demonstrated that HOXC‐AS3 is predominantly localized in the cytoplasm (Figure 4A,B). MIR22HG improves ANGPTL4 mRNA stability by recruiting YTHDC1 [24]. Multiple databases predicted that HOXC‐AS3 could bind to YTHDC1 (Figure 4C). Further analysis confirmed that HOXC‐AS3 can bind to YTHDC1 (*p* < 0.01, Figure 4D,E). Further database predictions identified downstream mRNAs that can be bound by YTHDC1 (Figure 4F). Among them, DLG4 expression is downregulated in CRC [19]. Similarly, it is assumed that DLG4 may be a downstream mechanism of HOXC‐AS3. RIP analysis revealed that YTHDC1 enriched more DLG4 mRNA (*p* < 0.01, Figure 4G). DLG4 expression was decreased in HCT116 and SW480 cells compared to that in NCM460 cells (*p* < 0.01, Figure 4H,I), and DLG4 expression was increased in the si‐KDM5C group compared to that in the si‐NC group (*p* < 0.01, Figure 4J,K). Furthermore, downregulation of HOXC‐AS3 significantly inhibited DLG4 expression, while overexpression of YTHDC1 promoted DLG4 expression (*p* < 0.01, Figure 4J–M). Moreover, downregulation of HOXC‐AS3 reduced DLG4 mRNA stability, while overexpression of YTHDC1 improved DLG4 mRNA stability (*p* < 0.01, Figure 4N). In summary, HOXC‐AS3 recruits YTHDC1 to promote DLG4 mRNA stability, therefore increasing DLG4 expression.

### 3.5 Downregulation of DLG4 Alleviates the Inhibitory Effect of KDM5C Downregulation on CRC Cell Proliferation

**FIGURE 5:**
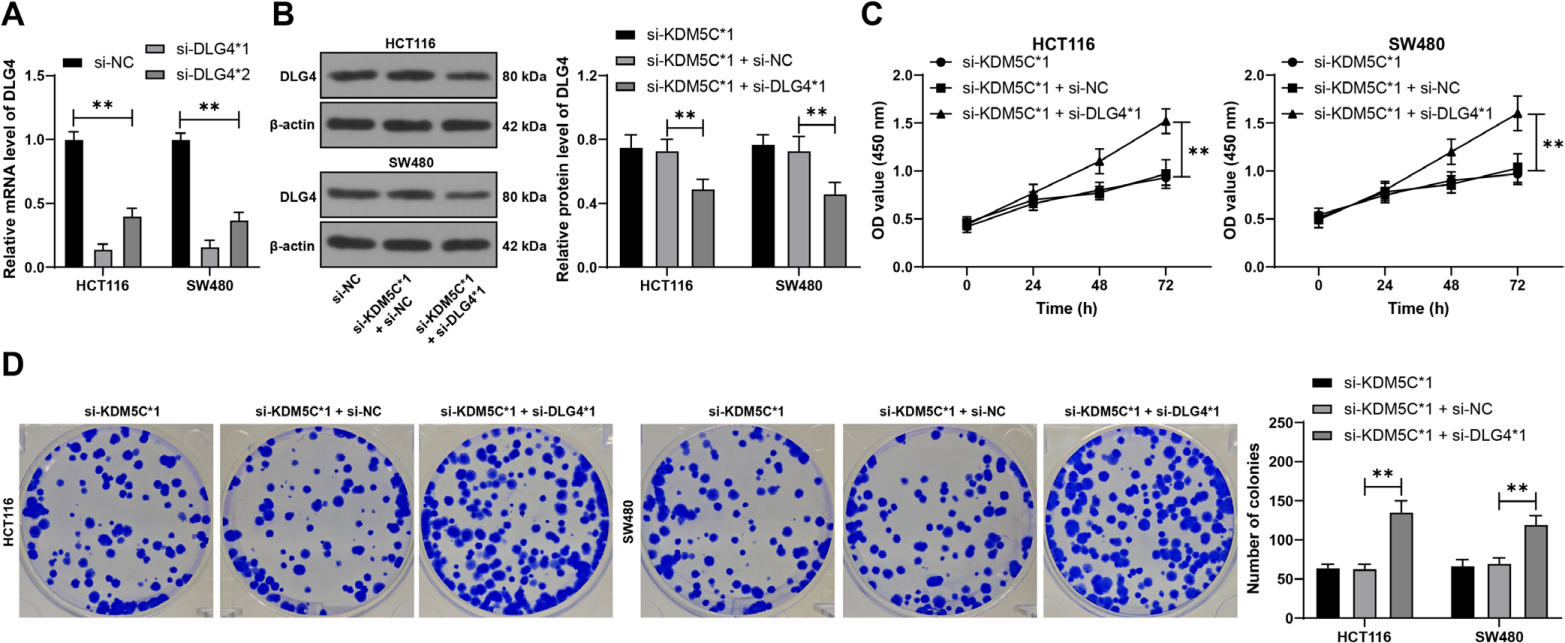
Alleviation of the inhibitory effect of KDM5C downregulation on CRC cell proliferation through downregulation of DLG4. DLG4 siRNA (si‐DLG4) was transfected into HCT116 and SW480 cells, with transfection of NC siRNA (si‐NC) as a negative control. (A, B) RT‐qPCR and Western blot were used to detect the expression of DLG4 in cells. (C) Cell proliferation was assessed using CCK‐8 assay. (D) Colony formation assay was performed to assess cell proliferation. The experiments were independently repeated three times, and the data are presented as the mean ± standard deviation. Multiple group comparisons in panels A–D were analyzed using two‐way ANOVA, followed by Tukey's multiple comparisons test. **p < 0.01.

To validate the role of DLG4 in HOXC‐AS3/KDM5C‐regulated CRC cell proliferation, the DLG4 expression in cells was downregulated and combined experiments were carried out using si‐DLG4*1 and si‐KDM5C*1, which showed improved transfection efficiency (*p* < 0.01, Figure 5A,B). Following the downregulation of DLG4, cell proliferation was increased (*p* < 0.01, Figure 5C), and colony formation was increased (*p* < 0.01, Figure 5D), thus alleviating the inhibitory effect of KDM5C downregulation on CRC cell proliferation.

## 4 Discussion

**FIGURE 6:**
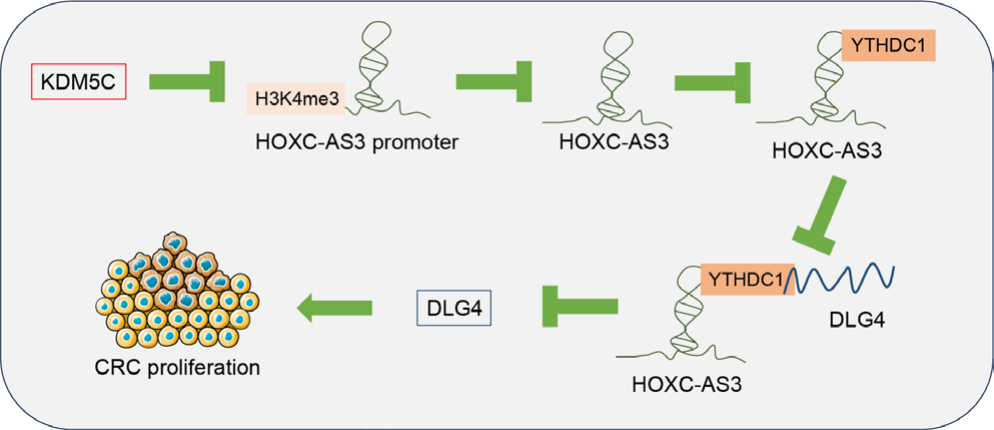
Mechanism of KDM5C in CRC cell proliferation. KDM5C‐mediated demethylation of H3K4me3 inhibits HOXC‐AS3 expression, reducing the recruitment of YTHDC1 by HOXC‐AS3, therefore decreasing DLG4 expression and ultimately promoting CRC cell proliferation.

Histone methylation is a crucial epigenetic modification involved in the development and progression of CRC and is considered to have translational potential for clinical applications [25]. KDM5C, a histone demethylase, mediates changes in chromatin structural remodeling in tumors and has both stimulatory and inhibitory effects on various cancer cell types [26]. In this study, upregulation of KDM5C in CRC cells was confirmed, and its oncogenic role was linked to KDM5C‐mediated demethylation of H3K4me3, which suppressed HOXC‐AS3 and decreased DLG4 mRNA stability and expression (Figure 6).

Growing evidence suggests a strong link between KDM5C dysregulation and CRC progression. KDM5C is overexpressed in CRC cells and clinical samples, where it mediates the demethylation of H3K4me3 on the PFDN5 promoter, inhibiting its transcription and promoting the malignant phenotype of CRC [9]. KDM5C suppresses METTL14 transcription by demethylation of H3K4me3, thus promoting CRC cell metastasis, migration, and invasion [10]. Overexpression of KDM5C interacts with downstream H3K4me3 marks on FBXW7 to suppress FBXW7 expression and lead to the accumulation of c‐Jun protein, resulting in CRC cell proliferation and poor overall survival [7]. The results showed that KDM5C was overexpressed in CRC, which aligns with this evidence. KDM5C decreased the enrichment of H3K4me3 on the HOXC‐AS3 promoter, thus enhancing CRC cell proliferation. These findings may offer a theoretical foundation for identifying downstream lncRNAs of KDM5C and broaden the potential of KDM5C as a therapeutic target for CRC.

Only one study addresses the role of HOXC‐AS3 in CRC progression. It found that CRC patients with low expression of HOXC‐AS3 had significantly lower overall survival rates, while overexpression of HOXC‐AS3 inhibited CRC cell invasion and migration [13]. Further study revealed that HOXC‐AS3 expression was low in CRC cells and its downregulation promoted cell colony formation under the influence of KDM5C‐mediated demethylation of H3K4me3. These findings provide further support for the anti‐carcinogenic role of HOXC‐AS3 in CRC. Targeting the upregulation of HOXC‐AS3 could offer a promising therapeutic strategy for CRC. However, the clinical impact of HOXC‐AS3 upregulation on prognosis requires further validation in future studies.

Subsequent database analysis predicted that HOXC‐AS3 could interact with YTHDC1. As an RNA‐binding protein, YTHDC1 can be recruited by lncRNAs to engage with target mRNAs post‐transcriptionally, improving their stability [27]. Another report has found that LINC00857 promotes CRC cell proliferation by recruiting YTHDC1 to improve the SLC7A5 mRNA stability in CRC cells [16]. Consistent with this prediction, this study was the first to confirm that HOXC‐AS3 recruits YTHDC1 to improve the stability and expression of DLG4 mRNA. Silencing DLG4 led to increased viability and proliferation of CRC cells.

Furthermore, DLG4 overexpression was found to suppress the oxidative pentose phosphate pathway flux and elevate reactive oxygen species levels in CRC cells, inhibiting tumorigenesis [16]. DLG4 was downregulated in colon adenocarcinoma patients and related to the prognosis [28]. However, studies investigating the specific role of DLG4 in CRC remain limited. Therefore, the mechanistic function of DLG4 in CRC should be further investigated in future experiments to identify novel therapeutic strategies.

This study has several limitations. First, mechanistic validation was limited to in vitro cellular models. Second, the analysis was confined to a single signaling axis without employing bioinformatics approaches to explore broader downstream pathways of KDM5C. Third, key processes such as metastasis and apoptosis in CRC cells remain unaddressed. Fourth, the potential involvement of other downstream miRNAs of HOXC‐AS3 and additional RNA‐binding proteins regulating the HOXC‐AS3/DLG4 axis has yet to be investigated. Lastly, the functional role of YTHDC1 as an m6A reader in CRC progression warrants more comprehensive exploration. The role of YTHDC1 in regulating CRC cell functions remains unexplored. Future studies will investigate whether YTHDC1‐mediated mechanisms influence metastasis and apoptosis in CRC cells. Moreover, clinical samples should be included to further elucidate the regulatory role of YTHDC1 in stabilizing DLG4, potentially offering novel theoretical insights for CRC therapy.

## 5 Conclusion

In conclusion, KDM5C‐mediated H3K4me3 demethylation suppresses HOXC‐AS3 expression, limiting its ability to recruit YTHDC1, subsequently leading to reduced DLG4 expression and enhanced CRC cell proliferation. These findings fill a critical gap in understanding how KDM5C‐driven epigenetic regulation impacts lncRNA expression in CRC, and they offer a novel theoretical foundation for considering KDM5C as a potential therapeutic target in CRC.

## Conflicts of Interest

The authors declare no conflicts of interest.

## Data Availability

The data that support the findings of this study are available from the corresponding author upon reasonable request.
